# Unraveling cardiolipin-induced conformational change of cytochrome *c* through H/D exchange mass spectrometry and quartz crystal microbalance

**DOI:** 10.1038/s41598-020-79905-8

**Published:** 2021-01-13

**Authors:** Sin-Cih Sun, Hung-Wei Huang, Yi-Ting Lo, Min-Chieh Chuang, Yuan-Hao Howard Hsu

**Affiliations:** 1 Department of Chemistry, Tunghai University, Taichung, Taiwan; 2 Department of Environmental Science and Engineering, Taichung, Taiwan; 3 Biological Science Center, Tunghai University, Taichung, Taiwan

**Keywords:** Biochemistry, Chemistry

## Abstract

Cardiolipin (CL), a crucial component in inner mitochondrial membranes, interacts with cytochrome *c* (cyt *c*) to form a peroxidase complex for the catalysis of CL oxidation. Such interaction is pivotal to the mitochondrial regulation of apoptosis and is affected by the redox state of cyt *c*. In the present study, the redox-dependent interaction of cyt *c* with CL was investigated through amide hydrogen/deuterium exchange coupled with mass spectrometry (HDXMS) and quartz crystal microbalance with dissipation monitoring (QCM-D). Ferrous cyt *c* exhibited a more compact conformation compared with its ferric form, which was supported by the lower number of deuterons accumulated and the greater amplitude reduction on dissipation. Upon association with CL, ferrous cyt *c* resulted in a moderate increase in deuteration, whereas the ferric form caused a drastic increase of deuteration, which indicated that CL-bound ferric cyt *c* formed an extended conformation. These results were consistent with those of the frequency (*f*) − dissipation (*D*) experiments, which revealed that ferric cyt *c* yielded greater values of |Δ*D*/Δ*f*| within the first minute. Further fragmentation analysis based on HDXMS indicated that the effect of CL binding was considerably different on ferric and ferrous cyt *c* in the C-helix and the Loop 9–24. In ferric cyt *c*, CL binding affected Met80 and destabilized His18 interaction with heme, which was not observed with ferrous cyt *c*. An interaction model was proposed to explain the aforementioned results.

Cytochrome *c* (cyt *c*) is a heme protein located in the mitochondrial intermembrane space in eukaryotic cells^1^. The negatively charged cardiolipin (CL)^2^ reacts with approximately 15% of cyt *c* under homeostatic conditions^3,4^. Oxidative stress induces peroxidation of CL to inhibit electron transport and alter mitochondrial bioenergetics^5–7^. Such CL peroxidation changes the integrity of inner mitochondrial membranes and affects the interactions between CL and cyt *c*^3,8–10^. These interactions facilitate unfolding and further activation of cyt *c* to catalyze CL peroxidation^11–13^ .The disruption of this protein–lipid complex triggers cyt *c* release from mitochondria and eventually leads to programmed cell death^14,15^.

Upon binding with CL, cyt *c* exhibits an active but less compact structure^16^. The CL binding also disturbs the interactions of Met80 with heme to unfold cyt *c* and therefore elevates its peroxidase activity. Three CL binding sites on cyt *c* have been reported, namely the ionic binding site A as well as the nonionic binding C- and L-sites^17,18^. Lys72, Lys73, and Lys79 residues of site A, next to Met80, are critical for CL recognition and necessary for the peroxidase activity^19^. CL on the liposome inserts a fatty acyl chain into the surface cleft between residues 67–71 and 82–85 on cyt *c*, which can be disrupted by Arg91 mutation^20^. Time-resolved FRET analysis of CL-bound cyt *c* revealed that the unfolding of cyt *c*, particularly on the C-terminus, increases its peroxidase activity^11^.

The redox state of the heme determines the affinity of cyt *c* to electron transport chain complex III and complex IV in mitochondria^21–23^ Ferric cyt *c* tends to associate with complex III, but ferrous cyt *c* favors binding to complex IV. The oxidation state of heme affects the conformation of cyt *c* and the affinity to the electron transport complexes. Several studies have suggested that the change of the charge state^23,24^ or redox potential^21,25,26^ triggers the conformational change of cyt *c*. Notably, the affinity changes toward electron transport complex did not drastically change the crystal structures^21,25^. In addition, the redox state of cyt *c* has been proposed to regulate cell growth. Ferric cyt *c* drives the cells toward the proapoptotic state and ferrous cyt *c* drives the stressed cells toward the prosurvival state^27^. Although both ferric and ferrous cyt *c* form complexes with CL^28^, whether different oxidative states of free cyt *c* affect their protein–lipid interactions to release cyt *c* during apoptosis has not yet been completely established.

We aimed to understand how the redox state of cyt *c* affects its interactions with CL. Amide hydrogen/deuterium exchange coupled with mass spectrometry (HDXMS) measures the rate of hydrogen/deuterium exchange on the peptide amide bond and has been widely used to analyze the interface of protein–protein interactions^29^, protein–DNA interactions^30^, protein–phospholipid interactions^31–34^, protein conformational changes^35^, and protein dynamics^36^. Quartz crystal microbalance with dissipation monitoring (QCM-D) is useful for determining the structural change of macromolecules^37–41^. Because the interactions between CL and cyt *c* can be electrostatic, hydrophobic, or of other types, determination of the exact conformation of the CL − cyt *c* association is difficult^13,42,43^. While NMR has been performed to study the interaction mechanism between cytochrome and lipid bilayers^44,45^, in the present study, we used QCM-D and HDXMS to measure and differentiate the structural change of both ferric and ferrous cyt *c* caused by CL binding. The information obtained from these two analytical methods provided an explanation of the interaction mechanism from global and fragmentary perspectives.

## Results

### Characterizations of cyt *c* and the CL-containing lipid membrane

The absorption spectra (Supplementary Fig. S1) revealed that ferric cyt *c* was reduced to the ferrous state through treatment with ascorbate. The Soret band (415 nm) of ferrous cyt *c* was red-shifted with respect to ferric cyt *c*. In addition, Q bands, residing in the range of 500–600 nm, transited from a broad absorption wave (maximum at 530 nm) to two discrete peaks (520 and 550 nm), verifying redox transition from ferric to ferrous states^46,47^. A successful synthesis of the CL/DPPC liposome was confirmed using NAO labeling, which specifically absorbed with CL (Supplementary Fig. S2). The fluorescence from the CL/DPPC-coated SiO_2_ surface was considerably greater than those of DPPC-coated and bare SiO_2_, which confirmed that CL was assembled with DPPC.

### Global hydrogen/deuterium exchange

**Figure 1:**
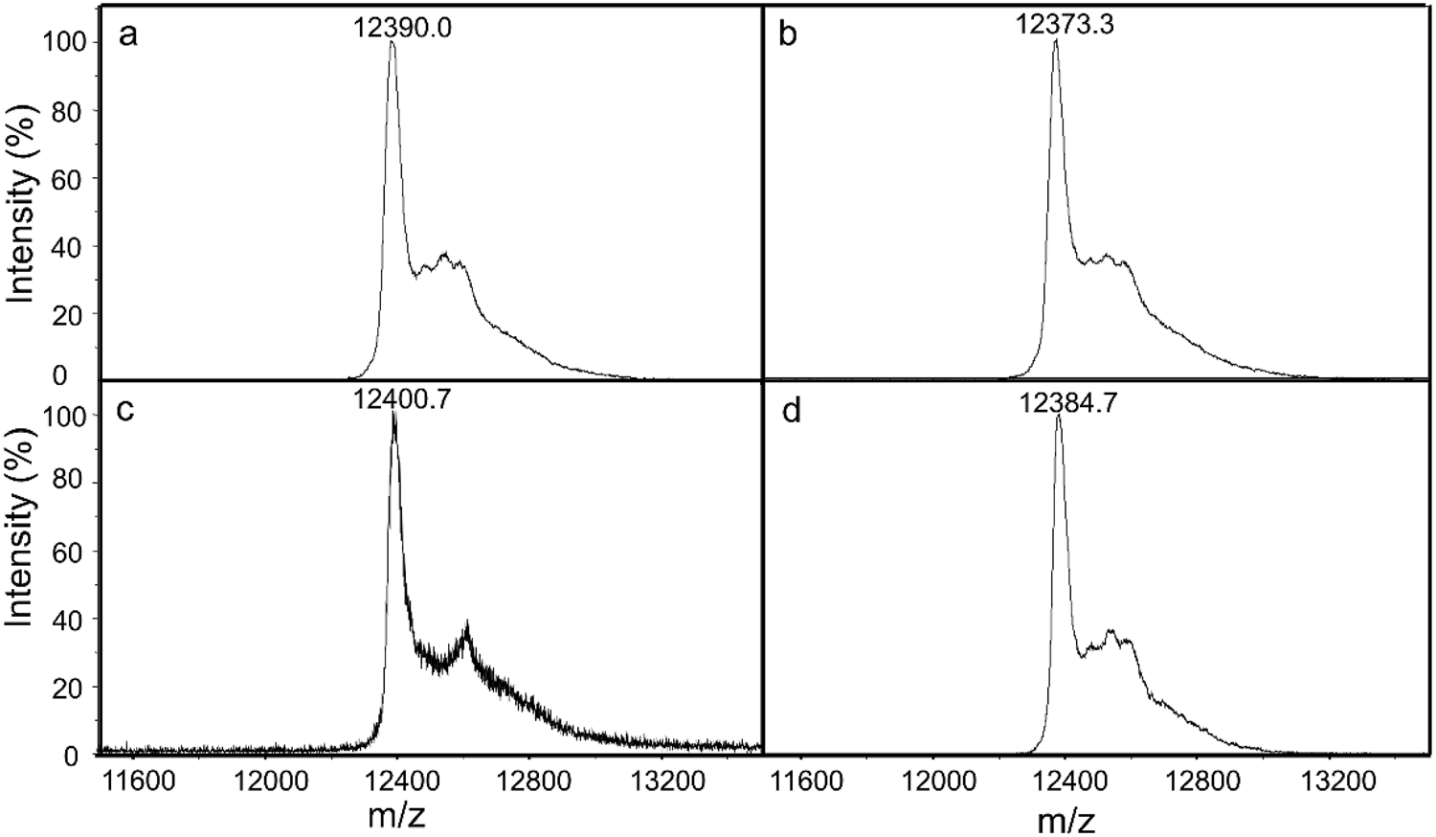
Mass spectra of pristine ferric (**a**), pristine ferrous (**b**), CL-bound ferric (**c**), and CL-bound ferrous (**d**) cyt *c* after 50-min deuteration. The CL-bound cyt *c* was prepared by incubating cyt *c* with a CL/DPPC liposomal solution at 30 °C for 10 min (see “Experimental” section for details).

The global H/D exchange of ferric and ferrous cyt *c* and their interaction with CL was analyzed using MALDI-TOF. Hypothetically, CL binding may induce conformational change on cyt *c* to yield varying levels of deuteron incorporation. As expected, ferric and ferrous cyt *c* exhibited similar masses of *m/z* = 12,344.0 and 12,344.1 Da, respectively (Supplementary Fig. S3). After 50 min of deuteration, ferric cyt *c* retained 46.0 deuterons and the mass shifted to 12,390.0 Da (Fig. 1A). However, ferrous cyt *c* accumulated 29.1 deuterons and the mass shifted to 12,373.2 Da (Fig. 1B). The greater extent of deuteration indicated that ferric cyt *c* held a less compact structure. These results implied the redox state of hemes was relevant to a perturbation on the Fe coordination with surrounding residue such as Met 80, leading to a loosen structure. Furthermore, the CL-bound cyt *c* exhibited an enhanced extent of deuteration, in which 56.7 and 40.6 deuterons were incorporated in ferric and ferrous cyt *c*, respectively (Fig. 1C,D). This result indicated that CL binding loosens the structure of cyt *c* in both ferric and ferrous states and potentially causes a substantially conformational change or a structural extension.

### Dynamic interaction monitored using QCM-D

**Figure 2:**
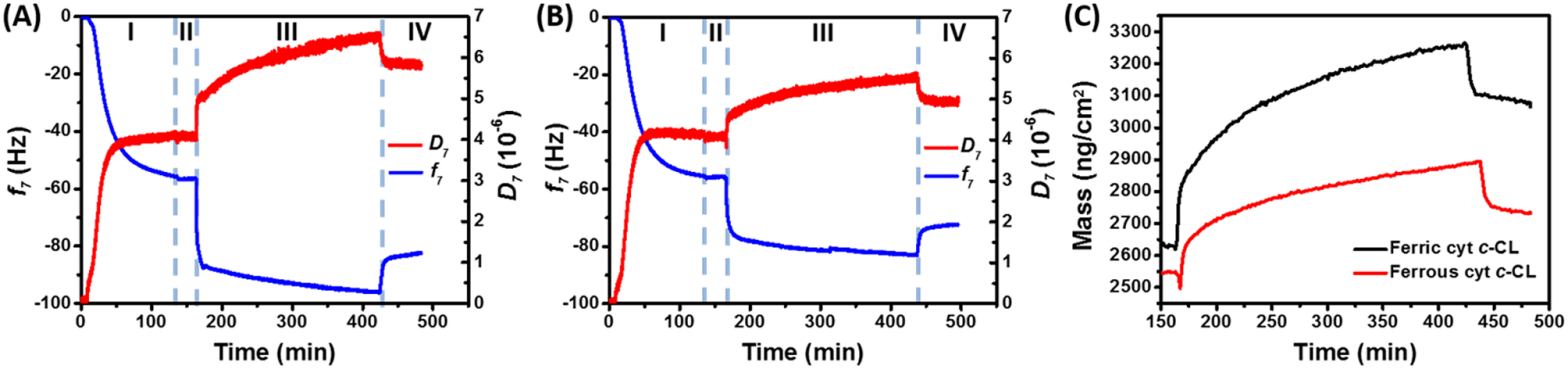
Frequency (blue) and dissipation (red) shifts (7th overtone) of a SiO_2_ QCM-D sensor. The sensor was sequentially exposed to the CL/DPPC liposomal solution (Section I), Tris–HCl buffer (Section II), cyt *c* solution (Section III), and Tris–HCl buffer (Section IV). (**A**) and (**B**) correspond to ferric and ferrous cyt *c*, respectively. (**C**) The masses of the adsorbed ferric (black) and ferrous (red) cyt *c* were estimated based on the Kelvin–Voigt model. Concentration of CL/DPPC liposome: 230 μM; concentration of cyt *c*: 50 μM.

Compared with the end-point analysis of the global deuteration of cyt *c*, a dynamic study is capable of offering insight into kinetics of the interaction between cyt *c* and CL. We performed QCM-D to analyze the in situ binding behavior. A schematic summarizing the analytical procedures (Sections I–IV) is depicted in Supplementary Fig. S4. The corresponding frequency (*f*) and dissipation (*D*) on the SiO_2_ sensor surface were recorded (Fig. 2A,B). As depicted in Section I of Fig. 2, *f* shifted to approximately − 56.52 Hz (Fig. 2A) and − 56.22 (Fig. 2B) with a *D* of 4.07 × 10^−6^ (at the 7th overtone), which indicated that the assembly of the CL/DPPC lipid layer on the SiO_2_ surface was reproducible. The subsequent exposure to the Tris–HCl buffer altered both Δ*f* and Δ*D* minimally (Section II). Crucially, a sequential exposure of the supported CL/DPPC membrane to cyt *c* substantially increased and decreased *f* and *D*, respectively. With the equilibration to the Tris–HCl buffer, the *f* of ferric cyt *c* shifted to − 82.50 Hz and *D* to 5.85 × 10^−6^ (Section IV, Fig. 2A). These shifts were considerably greater than those yielded by ferrous cyt *c* (− 72.50 Hz on *f* and 4.93 × 10^–6^ of *D*), which indicated that ferric, rather than ferrous, cyt *c* exhibited stronger affinity with CL. Furthermore, the greater dissipation shift (Δ*D*) was presumably attributed to an extensional structure of the ferric cyt *c*-bound CL/DPPC complex. This result was consistent with that observed in global H/D exchange experiments, in which 56 and 40 deuterons were retained in ferric and ferrous cyt *c*, respectively (Fig. 1).

On the basis of the change in *f* and *D*, the hydrated mass and shear modulus characteristic of the CL/DPPC-supported lipid membrane, cyt *c*-bound CL/DPPC complex, and difference representing net cyt *c* were estimated based on the Kelvin–Voigt model (Table 1). Accordingly, the masses of the ferric and ferrous cyt *c* bound with CL/DPPC were estimated to be 455 and 185 ng/cm^2^, respectively, which suggested that ferric—rather than ferrous—cyt *c* exhibited high affinity with CL (Fig. 2C). In addition, dissipation change data were used to estimate the shear modulus of species in the layered format. Although the CL/DPPC lipid membrane exhibited a shear modulus of approximately 6.334 × 10^3^ Pa, the interaction of cyt *c* with such lipid layers increased the shear modulus. Because the value of the shear modulus was regarded as a measure of stiffness, the result revealed that cyt *c* rendered the cyt *c*–CL/DPPC complex more rigid compared with bare CL/DPPC. In particular, ferrous cyt *c* enhanced the stiffness more (shear modulus increase of 4.325 × 10^3^ Pa, which was greater than 2.883 × 10^3^ Pa of ferric cyt *c*).

**Table 1:** Mass and shear modulus of the CL/DPPC supported lipid membranes, cyt *c*-bound CL/DPPC complex, and cyt *c* (difference between the former two).

	CL/DPPC	Ferric cyt *c*-CL/DPPC	Ferric cyt *c*	Ferrous cyt *c*-CL/DPPC	Ferrous cyt *c*
Mass (ng/cm^2^)	2585	3080	455.0	2730	185.0
Shear modulus (× 10^3^ Pa)	6.334	9.217	2.883	10.83	4.325

The values were estimated based on the Kelvin–Voigt model.

**Figure 3:**
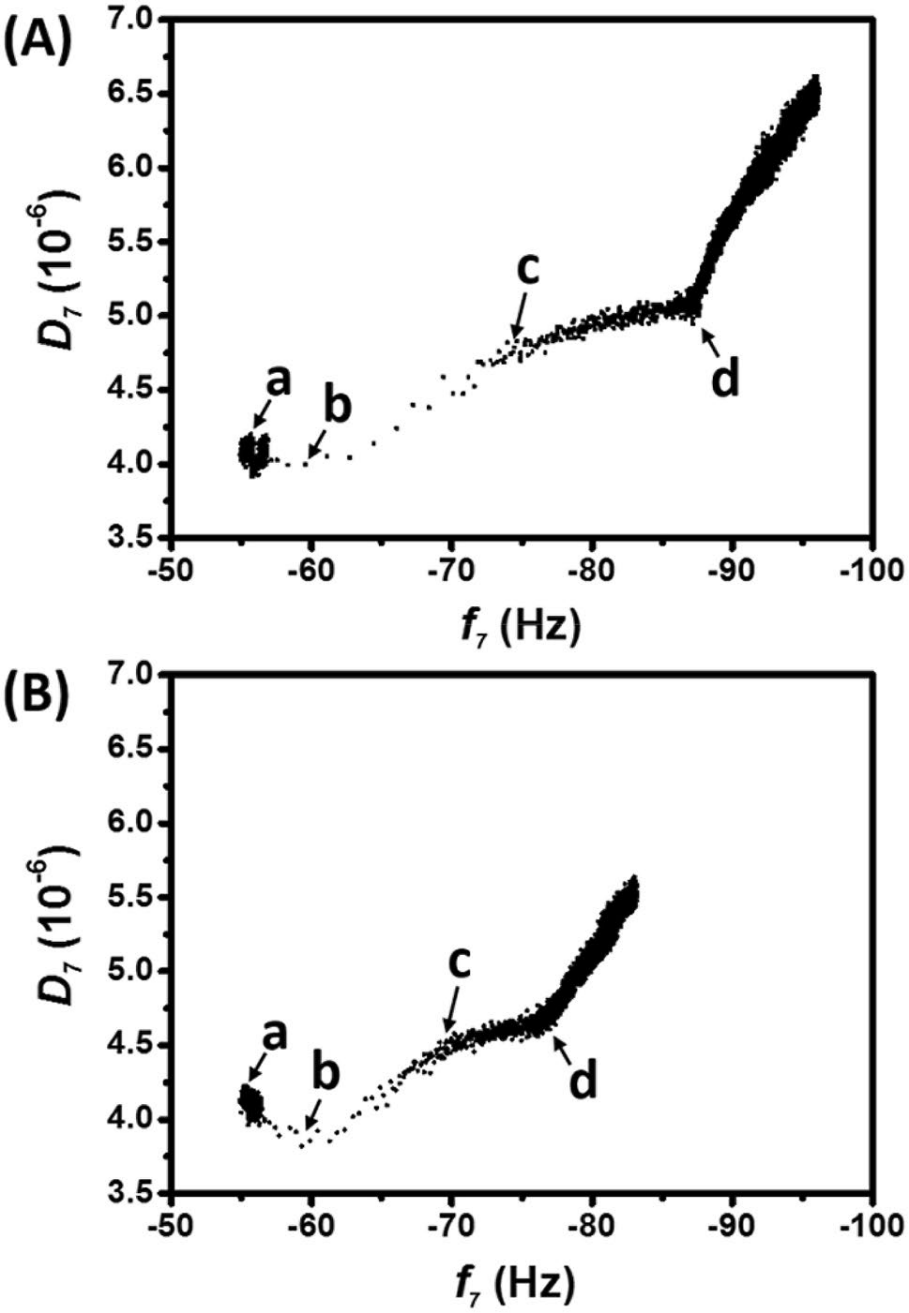
*D*–*f* curves of ferric (**A**) and ferrous (**B**) cyt *c*–CL interaction. The *D* values (7th overtone), recorded in Section III of Fig. 2A,B, were plotted against the corresponding *f*. The **a** and **b** denote that the onset of the CL/DPPC supported layer was in contact with cyt *c* and the state with the smallest *D*, respectively. The **c** and **d** indicate the states at which |Δ*D*/Δ*f*| changed.

Section III was further analyzed to understand the dynamic interaction between cyt *c* and CL/DPPC. The *D* values, recorded during Section III of Fig. 2A,B, were plotted against the corresponding *f* (Fig. 3). Accordingly, the |Δ*D*/Δ*f*| ratio was calculated to determine the structural relaxation of the cyt *c*-bound CL/DPPC complex caused by the per unit mass increase of cyt *c*. In both the ferric (Fig. 3A) and ferrous (Fig. 3B) cyt *c* cases, four |Δ*D*/Δ*f*| values were observed throughout the increasing *f* curve, which were represented in phases of **a** → **b**, **b** → **c**, **c** → **d**, and beyond **d** (Fig. 3). The point **a** denotes the onset at which the CL/DPPC lipid of supported layer was exposed to cyt *c*. *D* decreased with the increase in *f* until point **b**, the state with the smallest *D*. The **a** → **b** period was short (15 and 28 s for ferric and ferrous cyt *c*, respectively). However, the |Δ*D*/Δ*f*| of ferrous cyt *c* was greater than that of ferric cyt *c*, which indicated that ferrous cyt *c* altered the CL/DPPC lipid layer at greater amplitude, presumably because of the higher shear modulus (rigidity) of ferrous cyt *c* (Table 1). Subsequently, *D* increased with the increase in *f* at a constant |Δ*D*/Δ*f*| from points **b** to **c**, after which |Δ*D*/Δ*f*| changed. Ferric and ferrous cyt *c* reached point **c** at approximately 60 and 121 s, respectively. Cyt *c* partially inserted into the CL/DPPC lipid membranes and the structural extension of cyt *c* disrupted the order of the lipid layer within 1 min^11,48,49^. Analysis of Δ*f* (within 1-min) revealed a higher interaction rate of ferric cyt *c* (0.304 Hz s^−1^) and (0.173 Hz s^−1^ of ferrous cyt *c*). Furthermore, ferric cyt *c* yielded a greater |Δ*D*/Δ*f*| (4.141 × 10^−8^ Hz^−1^) compared with the 2.635 × 10^−8^ Hz^−1^ of ferrous cyt *c* within 1 min, which indicated that single ferric cyt *c* not only exhibited higher CL/DPPC affinity but also rendered a complex and extended structure. Compared with the **b** → **c** period, the |Δ*D*/Δ*f*| values in the **c** → **d** period were smaller. However, the |Δ*D*/Δ*f*| values observed beyond point **d** were greater than those in the **b** → **c** duration. We proposed that point **d** was the end of the first molecular interaction layer. The gradual decline on |Δ*D*/Δ*f*| from **c** to **d** indicated that the late-interacting cyt *c* had less of an effect on the structure of the cyt *c*–CL/DPPC complex because of the reduced negative charge of CL on the surface. The high |Δ*D*/Δ*f*| values beyond point **d** were attributed to the interaction beyond the first molecular layer of interaction. After saturation in the first layer, the non-adsorbed cyt *c* exhibited physical adsorption instead of a specific electrostatic interaction with CL/DPPC. As such, the |Δ*D*/Δ*f*| represented the viscoelastic properties which were attributed to pristine cyt *c*. Overall, the |Δ*D*/Δ*f*| under the first layer (from points **a** to **d**) of the interaction was 3.457 × 10^−8^ and 2.718 × 10^−8^ Hz^−1^ for ferric and ferrous cyt c, respectively, which indicated a less dense structure of the ferric cyt *c*-bound CL/DPPC complex. This was consistent with the results of previous studies^11,49^.

### HDXMS of pepsin-digested cyt *c*

In addition to using HDXMS and QCM-D, we aimed to retrieve detailed regional information of the protein. The HDXMS data of the pepsin-digested ferric cyt *c* revealed that 55 peptides accounted for complete coverage of the protein primary sequence (Supplementary Fig. S5), which indicated that the HDXMS result could provide information in every residue. The deuteration level of the representative peptides in the specific regions of cyt *c* are presented under the primary sequence and mapped onto a NMR structure in Supplementary Fig. S6^23^. As mentioned earlier, heme is a Fe-containing ligand coordinating with Met80 and His18 in the cyt *c* structure. The His18 containing loop was stabilized by both His18 coordination and covalent bonding between the Cys14 and Cys17 thiols and heme (Supplementary Fig. S6C). We observed low deuteration levels in the loops 10–24, 25–32, and 35–46, which contained the L-site and a CL binding site composed of Lys22, Lys27, and His33, and were close to heme coordination residue His18. By contrast, the Met80-containing loop and 70 s Helix region (67–82) were deuterated to a relatively high level. Another CL binding region (A-site), containing Lys72, Lys73, Lys86, and Lys87, was located in this region. In addition, the contact regions of n-terminal and c-terminal helices, as well as the 50 s helix, were deuterated to high levels (Supplementary Fig. S6).

### Redox-dependent deuteration of cyt *c*

**Figure 4:**
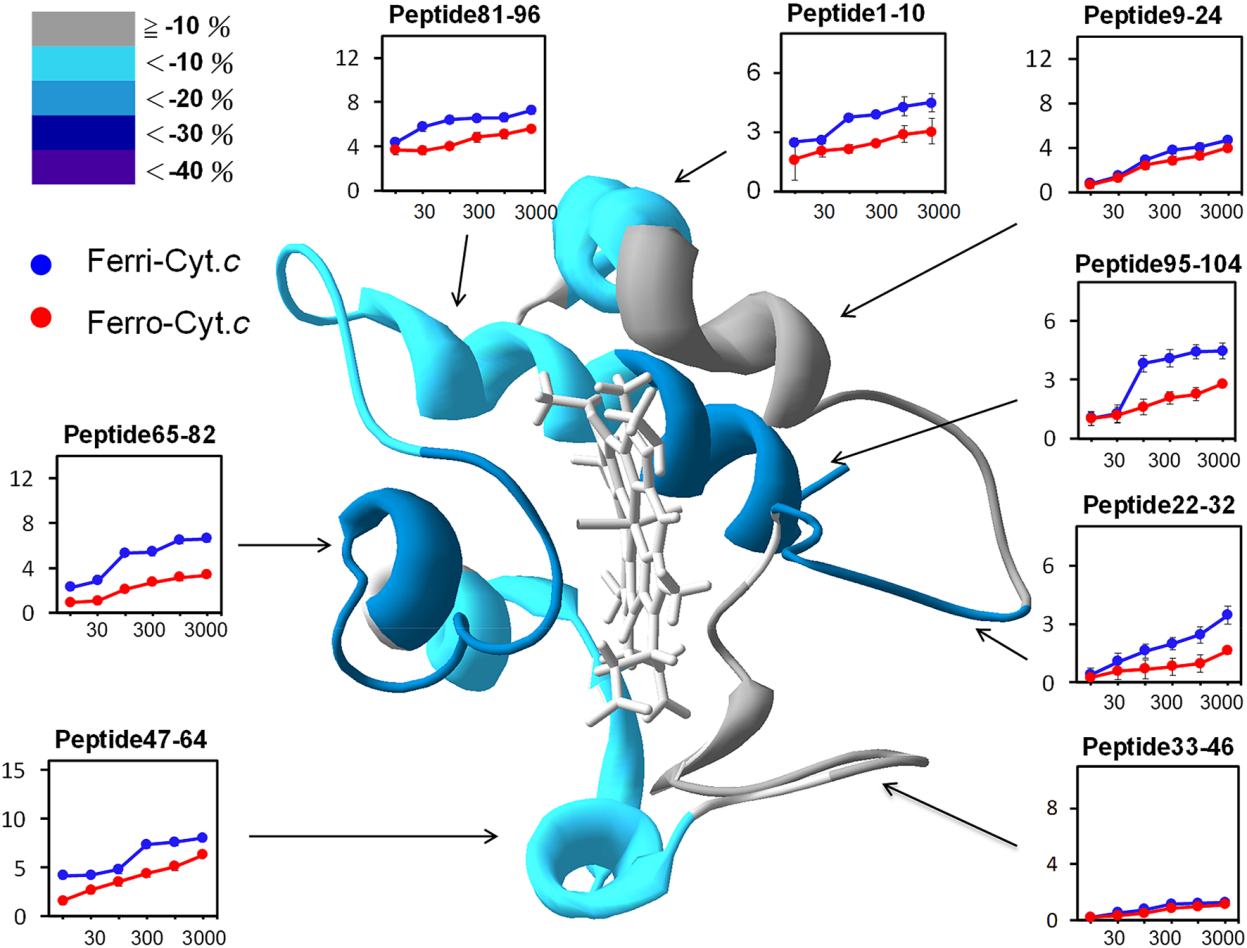
Deuteration level of ferric (blue) and ferrous (red) cyt *c* represented by peptide fragments. The difference in the deuteration level between ferric and ferrous cyt *c* is illustrated in percentage [(ferrous − ferric)/ferric × 100%] in different colors (scale shown in the top-left corner). The time-course deuterium number of each fragment related to the corresponding region of cyt *c* (the ribbon diagram shown in the center of the figure) was recorded.

To investigate the conformational change of cyt *c* induced by heme redox, an HDXMS analysis of ferrous cyt *c* was conducted. Compared with ferric cyt *c*, ferrous cyt *c* exhibited a lower (0–20% reduction) level of deuteration in most fragments (Fig. 4), which indicated a stable structure of the ferrous cyt *c*. The results were consistent with the higher shear modulus obtained through QCM-D. A detailed examination of the heme-proximity region revealed that the heme reduction decreased deuteration of regions 65–82, containing Met80. However, minimal changes occurred in regions 9–24, containing His18. The contact area between C-terminal regions 95–104 and N-terminal regions 1–10 exhibited considerable decreases in deuteration (approx. 50%). The disturbance of Fe-Met80 coordination had a limited effect on 60 s and 70 s helices. Given that no changes were observed in His18, the effects on the N-terminal helix (i.e., the deuteration decrease in this region) were probably affected by contact with the C-terminal helix. These results were consistent with those obtained using NMR^23^, in which two redox states were compared. The results proved that oxidation of the heme changed Fe-Met80 coordination and triggered a structural change to expose the heme molecule outward. Approximately 15% of the surface area of heme (III) became solvent accessible, which was higher than 7% of heme (II). This behavior highlighted the higher deuteration of ferric cyt *c* caused by the high solvent accessibility.

### HDXMS study of CL-bound cyt *c*

**Figure 5:**
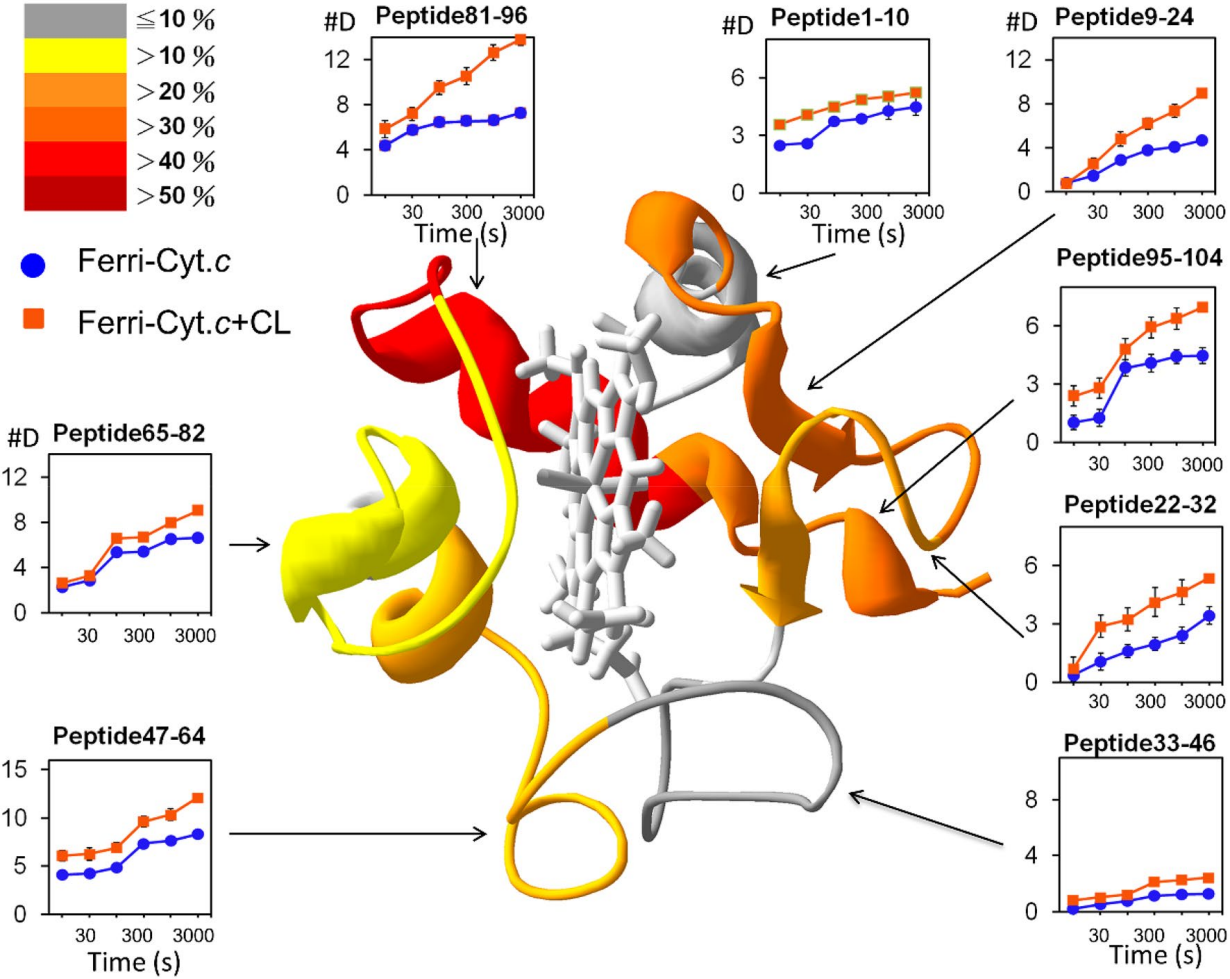
Deuteration level of ferric cyt *c* (blue) and the CL-bound ferric cyt *c* (orange) represented by peptide fragments. Enhancement of deuteration caused by CL binding is illustrated in percentages and displayed in different colors (scale shown in the top-left corner). The time-course deuterium number in each peptide fragment with the corresponding region of cyt *c* is presented (the ribbon diagram shown in the center of the figure).

HDXMS analyses were conducted to investigate the structural change caused by CL binding. Figure 5 displays the deuteration level of ferric cyt *c* (blue) and the CL-bound ferric cyt *c* (orange) as functions of the time period of deuteration and peptide fragments. Generally, the deuteration level increased by 1–50% upon CL binding. The C-terminal helix in particular exhibited drastic increases near the thiol side of heme, including a 46.2% increase in regions 81–96, part of the A-site, and 32.5% increase in regions 95–104. The Loop 9–24 and Loop 22–32 on the L-site exhibited increases of 30.6% and 26.1%, respectively. Because the two significantly affected regions were not physically in contact with each other, CL binding presumably distorted both regions simultaneously. The increase of cyt *c* deuteration indicated that CL binding did not block the solvent accessibility in the binding regions, but increased the flexibility of cyt *c* with an extended conformation.

**Figure 6:**
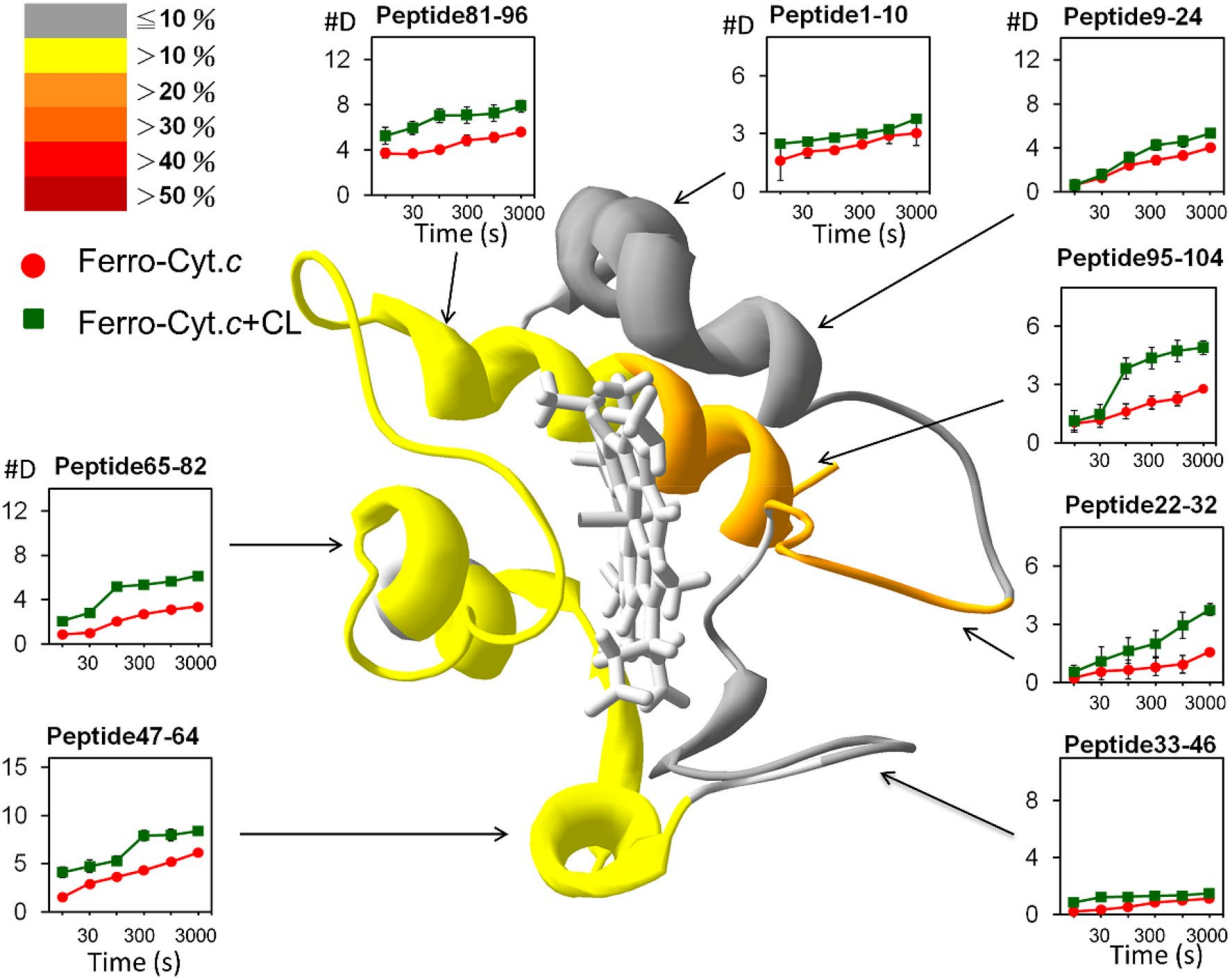
Deuteration level of ferrous cyt *c* (red) and the CL-bound ferrous cyt *c* (green) represented by peptide fragments. Enhancement of deuteration caused by CL binding is illustrated in percentages and displayed in different colors (scale is shown in the top-left corner). The time-course deuterium number in each peptide fragment with the corresponding region of cyt *c* is presented (the ribbon diagram shown in the center of the Figure).

By contrast, the deuteration increase of ferrous cyt *c* caused by CL binding was limited (Fig. 6), which could be attributed to a compact structure. A minimal change occurred in the deuteration level of the N-terminus and the His18-containing loop upon CL binding. Regions 9–24 in ferrous cyt *c* were stable and therefore CL binding did not affect this heme coordination region. The deuteration level changed substantially in the regions 95–104 (28.9%) at C-helix and Loop 22–32 (30.1%). Regions 47–64 and 65–82 exhibited minor changes at 16.5% and 21.6%, respectively. Overall, the deuteration-enhanced regions and amplitude were similar to those of ferric cyt *c*. Crucially, regions 9–24 exhibited a 30.6% change in ferric cyt *c*, compared with only 10.6% in ferrous cyt *c*, which indicated that CL binding disturbed heme coordination with His18 in ferric cyt *c*, but not in ferrous form. This also suggested that CL interacted with the A-site and L-site in ferric cyt *c*, but only with the A-site in ferrous form. An alternating current voltammetric study on a cyt *c*-immobilized electrode also supported this conclusion (Supplementary Fig. S7), in which the potential shift (24.1 mV) of ferric cyt *c* induced by CL binding was greater than 9.20 mV of ferrous cyt *c*. Furthermore, the redox potential of cyt *c*, principally acted by heme, shifted cathodically, which indicated a relatively facile electron transfer occurred upon CL binding.

## Discussion

The results are summarized as follows: (i) CL binding had a different effect on ferric and ferrous cyt *c* in C-helix and Loop 9–24. (ii) Loop 9–24, containing Fe coordination residue His18 and the thioether covalent bonding residues Cys14 and Cys17, stabilized the relative position of heme and the protein structure. (iii) CL interaction with ferric cyt *c* not only affected Met80 but also destabilized His18 interaction with heme, which was not observed in ferrous cyt *c*. (iv) The C-helix on ferric cyt *c* was close to full deuteration after 30 min of deuteration, which indicated a high exposure of this region; however, such behavior was not observed in ferrous cyt *c*. The structured extension of C-helix also highlighted the conclusion revealed by FRET^11^. (v) The contacted N-helix did not exceed a 10% difference, which indicated the interaction between the N-helix and C-helix remained intact in this extended conformation.

Although studies on the activation mechanisms of phospholipase A_2_ on membranes have indicated that membrane association and penetration mostly cause decreased deuteration^31,33,34^, Skinner et al. proved that H/D exchange depends primarily on the hydrogen bonding status of the amide hydrogen; only a minor contribution is from solvent accessibility^50,51^. Thus, the increasing deuteration revealed in the current study was probably because of the results of conformational changes and because of blocking solvent accessibility. Although either the insertion of cyt *c* into CL/DOPC^48,49^ or the extended CL anchorage accommodated into cyt *c*^52^ was proposed, our results suggested that cyt *c* did not, if any, only occurred in the very beginning association (within a couple seconds), substantially penetrate into the hydrophobic motif of liposome.

On the basis of the aforementioned results, a model was proposed. In a healthy cell, the mitochondrial intermembrane space full of protons is in an oxidizing state^53^. Therefore, protons compete with cyt *c* to associate with CL^54^. Free ferric cyt *c* can therefore transfer electrons from ETC III to ETC IV. Under oxidative stress or mitochondrial damage, the loss of proton gradient and electron transfer results in a low concentration of proton and ferrous cyt *c*. Without competition from proton and ferrous cyt *c*, ferric cyt *c* tightly associates with CL. CL binds to both the A- and L-sites of flexible ferric cyt *c* and triggers the structural extension of ferric cyt *c*, which further activates cyt *c*. Less compact and extended cyt *c* exposes the active site on the CL-liposomal surface to oxidize CL^55^. The oxidized CL inhibits the cyt *c* interacting with the mitochondrial inner membrane, leading to the release of cyt *c* from mitochondria and the subsequent apoptosis process.

## Methods

### Chemicals and materials

Cyt *c* (from equine heart), phosphatidylcholine (eggPC, from egg yolk), 3-Mercaptopropionic acid, 6-Mercaptohexanol, potassium chloride (KCl), sodium l-ascorbate, ethanol, 1-Octadecanethiol (ODT), trifluoroacetic acid (TFA), acetonitrile (ACN), and sinapinic acid were purchased from Sigma (St. Louis, MO). The lipids 1,2-dipalmitoyl-*sn*-glycero-3-phosphocholine (DPPC) and 1′,3′-bis[1,2-dioleoyl-*sn*-glycero-3-phospho]-glycerol (18:1, CL) were obtained from Avanti Polar Lipids (Alabaster, AL). Sodium dodecyl sulfate (SDS) was purchased from Merck (Darmstadt, Germany). Deuterium oxide (D_2_O, 99.9%) was obtained from Cambridge Isotope Laboratories (Tewksbury, MA). Immobilized pepsin on 6% agarose beads was purchased from Thermo Scientific (Waltham, MA). A protein assay dye reagent concentrate was purchased from Bio-Rad (Hercules, CA). All aqueous solutions were prepared using ultrapure water (18.2 MΩ cm) from a Milli-Q purification system (Millipore, Burlington, MA). A CL binding dye, 10-nonyl acridine orange (NAO), was obtained from Enzo Life Sciences (Farmingdale, NY).

### Preparation of CL/DPPC liposome

We adopted 20 mol% of (18:1)_4_CL/DPPC as the experimental system. Considering the critical micelle concentration (CMC) difference between DOPC and DPPC^56–58^, the DPPC liposome can be formed readily. Plus, lipids containing lower saturation of the fatty acyl chains were considered to exist in inner membrane contact sites^59^. Furthermore, the literature also showed that 20 mol% of DPPC/CL formed the most thermodynamically stable binary monolayer^60^. CL-containing liposome was prepared by dissolving DPPC (7.56 mg) and 18:1 CL (3.75 mg) at a molar ratio of 4:1 in chloroform in a glass vial. The chloroform was then evaporated using a rotary evaporator at 45 °C, leaving a liposomal gel film. The dried constituents were reconstituted with 1 mL of 100 mM KCl (for HDXMS) or 50 mM Tris–HCl (pH 7.0, for QCM-D), and sonicated at 60 °C for 30 min. The resulting liposome was sequentially extruded through a polycarbonate filter (100 nm, Whatman) to form liposomal capsules. Before HDXMS experiments, the CL/DPPC liposome was equilibrated in a 30 °C water bath for 30 min.

### Matrix-assisted laser desorption/ionization (MALDI-TOF) analysis

A gold-coated silicon wafer was used as the substrate in MALDI-TOF measurement. The wafer was treated with ODT (0.1 mM), which was then fixed on the MALDI target plates and precooled on ice. The target plate was customized to compensate for the thickness (1 mm) of the silicon wafer. The sample for MOLDI-TOF analysis was prepared by mixing 1 μL each of cyt *c* and sinapinic acid. The mixture was then spotted onto the cooled substrate and dried in a moderate vacuum. The dried sample was immediately analyzed using MALDI-TOF (Microflex, Bruker) at 4.2 × 10^−6^ bar, 70% laser energy (79 μJ), and 10 scan/sec for 30 s. The data were acquired and analyzed using the Mmass program.

### QCM-D measurement

A QCM-D instrument (Q-sense E1, Q-sense, Gothenburg, Sweden) was used to monitor the interaction between cyt *c* and CL. An SiO_2_-coated quartz crystal (QSX 303, Q-sense) sensor was first cleaned by immersion in 2% SDS for 30 min, followed by UV ozone treatment for 10 min, before being installed into the QCM-D chamber. To form lipid bilayers on the surface of the SiO_2_ sensors, a CL-containing liposomal solution (1 mg/mL, equilibrated in 50 mM Tris–HCl buffer, pH 7.4) was pumped (200 µL/min) over the SiO_2_ sensor surface for 2 h. Subsequently, the flow liquid was changed to 50 mM Tris–HCl buffer to remove excess liposomes. Then, either a ferric or ferrous cyt *c* solution (50 µM) was drawn (200 µL/min) over the CL-containing lipid bilayer for 4 h. Nonspecifically adsorbed cyt *c* was subsequently removed using the Tris–HCl buffer (50 mM, pH 7.4). QCM-D measurement was performed at overtones 1, 3, 5, 7, 9, 11, and 13 of a fundamental frequency in 5 MHz at 25 °C. The dynamic shifts on both frequency and dissipation were acquired. The hydrated mass and shear modulus were estimated using Q-tools (Biolin Scientific, Gothenburg, Sweden) software based on the Kelvin–Voigt model, with densities of the lipid membrane and protein of 1.1 and 1.35 g/cm^3^, respectively^61^.

### Preparation of deuterated samples

Prior to hydrogen/deuterium (H/D) exchange experiments, 60 μL of cyt *c* (16.6 mg/mL) was mixed with 40 μL of the prepared liposomal solution and incubated at 30 °C for 10 min. H/D exchange experiments were initiated by mixing 5 μL of the aforementioned mixture with 20 μL of 95% D_2_O buffer (125 mM Tris, 50 mM NaCl, pH7.5) to a final concentration of 76% D_2_O at pH 7.5 and 125 μM lipid vesicles. The samples were incubated at 23 °C for an additional 10, 30, 100, 300, 1000, or 3000 s. The deuterium exchange was quenched by adding 175 μL of TFA (0.1%). The samples were then immediately digested using pepsin-bound agarose beads on ice for 5 min and quickly vortexed every 30 s.

### Electrospray ionization (ESI)-MS/MS analysis

The pepsin-digested cyt *c* was manually injected and trapped by the Trap column (Optimize, 3 mm cartridge). The deuterated peptides were subsequently separated using a high-performance liquid chromatography C18 column (Biobasic 5 μm 50 mm × 1 mm, Thermo Scientific) and then sequentially analyzed through ESI–MS or ESI–MS/MS (Esquire 6000, Bruker, Billerica, MA). The gradient of the mobile phase was varied from 0% A solution (0.1% TFA) and 100% B solution (0.01% TFA, 80% ACN) to 100% A in 40 min at a flow rate of 0.1 mL/min. The buffer, column, and tubing were immersed in ice water as previously described^29^. Back exchange levels were calculated based on 24-h fully deuterated samples as previously reported^62^.

### Data analysis

An intensity threshold (5000-count) of the peptides was first filtered using Data Analysis 3.4 (Bruker Corporation). The sequence of the peptides was identified through MS/MS analysis in triplicate. Peptide identification was performed on an X!Tandem parser 1.7.7 followed by further manual examination of product ions. The peptides that were recognized more than twice and with an excellent ion match in the product ion spectrum were selected into the pool. HDexaminer 1.3 (Sierra Analytics) was used for the mass spectra analysis, which was similar to a previously described version^29^. The results were incorporated into HDexaminer, which retrieved the charges, sequences, and retention times from the mass data. The software evaluated the match between the experimental data and theoretical mass envelopes, and provided a score for each peptide fragment. Every mass envelope was further manually examined to ensure the mass envelope was identified correctly. The mass shifts at different time points of 0, 10, 30, 100, 300, 1000, and 3000 s were calculated individually. The deuteration level of each peptide was determined using the ratio of the incorporated deuteron number to the maximum possible deuteration number. Because of the fast off-exchange rate of the two N-terminal residues, those residues could not retain any deuterons after liquid chromatography and therefore were not included in the calculation.

## Conclusions

The differential conformational change of cyt *c* during CL-association was confirmed by the results from the QCM-D and HDXMS analyses. The data indicated that ferric cyt *c*, not ferrous cyt *c*, exhibited an extended and partially unfolded structure on the lipid surface. The domains of cyt *c* were not inserted into the liposome; if they did, it would likely occur at the initial stage for a few seconds. This was highlighted by the increasing deuteration and dissipation shift in the initial interaction. The CL molecule was proposed to be extracted out of the lipid aggregates to act as the substrate for a cyt *c*-induced peroxidation, which presumably caused the decreasing dissipation observed in the beginning stage of interaction (Section III).

## Supplementary Information

Supplementary Information.
