# Supplementary material for: Unraveling cardiolipin-induced conformational change of cytochrome c through H/D exchange mass spectrometry and quartz crystal microbalance
Source: Sci Rep. 2021 Jan 13;11:1090. doi: 10.1038/s41598-020-79905-8 (PMC7806790; doi:10.1038/s41598-020-79905-8)
Supplement: Supplementary file 1 — Supplementary Information. [file 41598_2020_79905_MOESM1_ESM.docx]

**Supplementary Information**

**Unraveling Cardiolipin-Induced Conformational Change of Cytochrome *c* Through H/D Exchange Mass Spectrometry and Quartz Crystal Microbalance**

Sin-Cih Sun^1^, Hung-Wei Huang^1^, Yi-Ting Lo^1^, Min-Chieh Chuang^1,2^* and Yuan-Hao Howard Hsu^1,3^*

^1^Department of Chemistry, Tunghai University, Taichung, Taiwan

^2^Department of Environmental Science and Engineering, Taichung, Taiwan

^3^Biological Science Center, Tunghai University, Taichung, Taiwan

*To whom correspondence should be addressed.

MCC: Phone, 886-4-23590121 ext. 32218; FAX, 886-4-23590426; E-mail, [mcchuang@thu.edu.tw](mailto:mcchuang@thu.edu.tw)

YHH: Phone, 886-4-23590121 ext. 32230; FAX, 886-4-23590426; E-mail, [howardhsu@thu.edu.tw](mailto:howardhsu@thu.edu.tw)

Submitted to ***Scientific Reports***

**Table of Contents**

**Figure S-1.** UV-Vis absorption spectra of cyt *c* 2

**Preparation of ferrous cyt *c*** 2

**Figure S-2.** Identification of CL on SiO_2_ sensor surface 3

**Figure S-3.** Mass spectra of cyt *c* measured by MALDI-TOF 4

**Figure S-4.** Schematic illustration of the investigation process using a SiO_2_ QCM-D sensor 5

**Figure S-5.** Peptide fragments of cyt *c* analyzed 6

**Figure S-6.** Ribbon diagram of ferric cyt *c* and its regional deuteration level 7

**Figure S-7.** AC voltammetry of cyt *c* immobilized on electrode 8

**
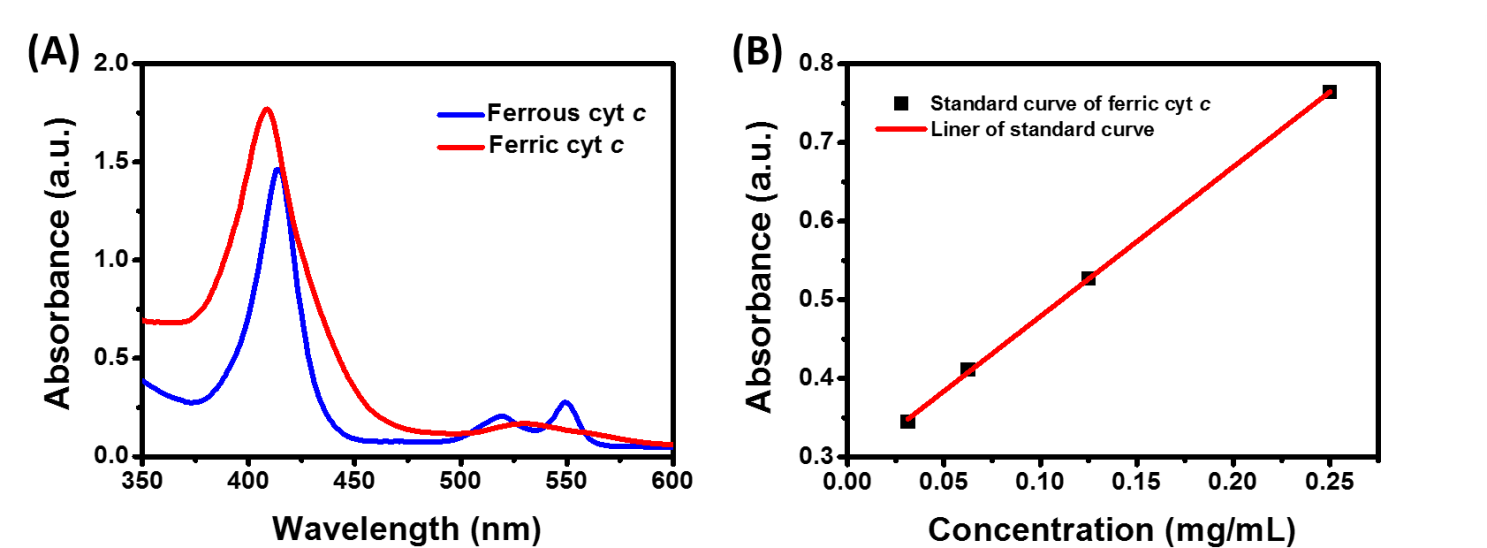
**

**Figure S-1.** (A) UV-Vis absorption spectra of ferric (red) and ferrous (blue) cyt *c*. (B) Calibration curve of ferric cyt *c* measured using Bradford assay.

**Preparation of Ferrous cyt *c*.** An aliquot (200 μL) of ferric cyt *c* (10 mg/ml in deionized water) was mixed with 200 μl of sodium ascorbate (100 mM) and incubated at 22 °C for 10 min to make ferric cyt *c* to ferrous form. The ferrous cyt *c* was collected by using a molecular weight cut-off nitrocellulose filter (3000 Da, Pall Corporation) under a centrifugation at 10,000 rpm. Ultimately the cyt *c* was replenished with deionized water with total volume of 180 μL. The resulting ferrous cyt *c* was characterized using UV-Vis spectrometer. Concentration of ferrous cyt *c* was analyzed with Bradford assay based on a calibration curve corresponding to ferric cyt *c*.


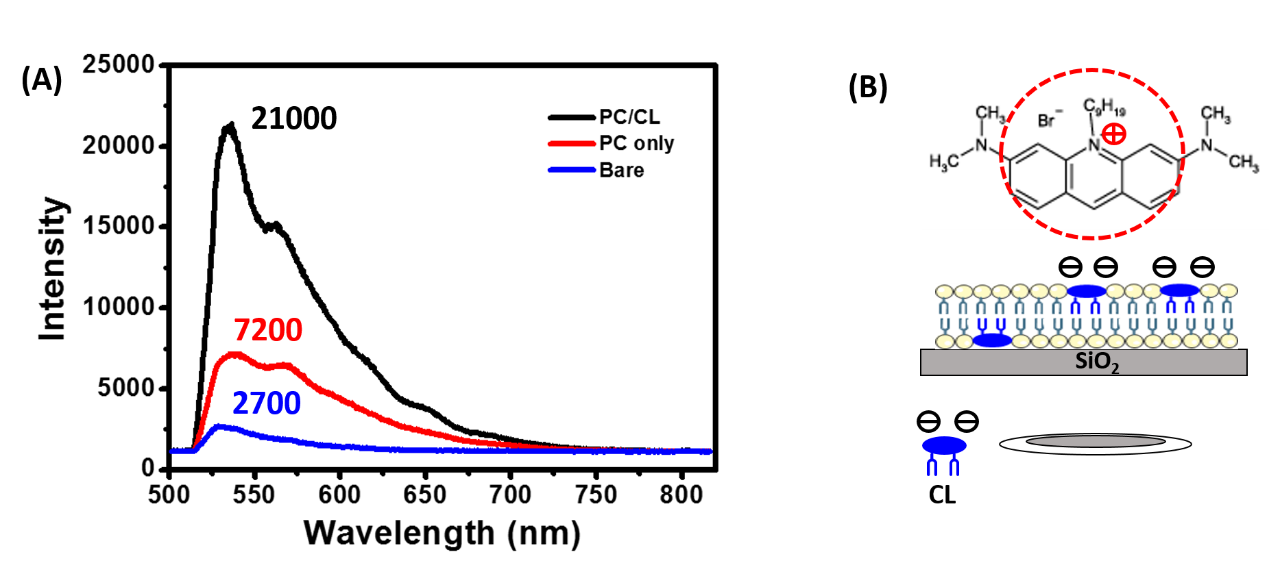


**Figure S-2.** (A) Fluorescent measurement (excitation wavelength at 496 nm) of bare (blue), DPPC- (red), and CL/DPPC- (black) coated SiO_2_ sensors. The sensors were stained with NAO before the measurement. (B) The schematic diagram of NAO-specific absorption with CL-containing lipid bilayer.

**Figure S-3.** Mass spectra of ferric (A) and ferrous (B) cyt *c* measured by MALDI-TOF.


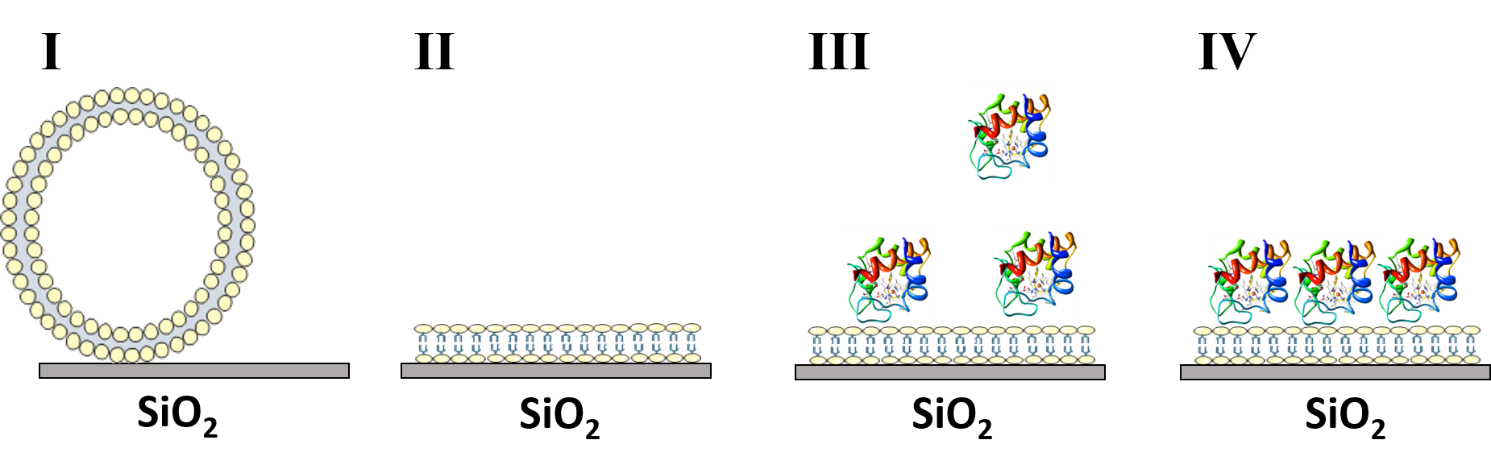


**Figure S-4.** Schematic illustration of the process investigating the binding between cyt *c* and CL/DPPC lipid membrane on a SiO_2_ QCM-D sensor. The sensor was exposed to the CL/DPPC liposomal solution (I), Tris-HCl buffer (II), the cyt *c* solution (III), and Tris-HCl buffer (IV) sequentially.

***H/D exchange of ferric cyt c***-

**
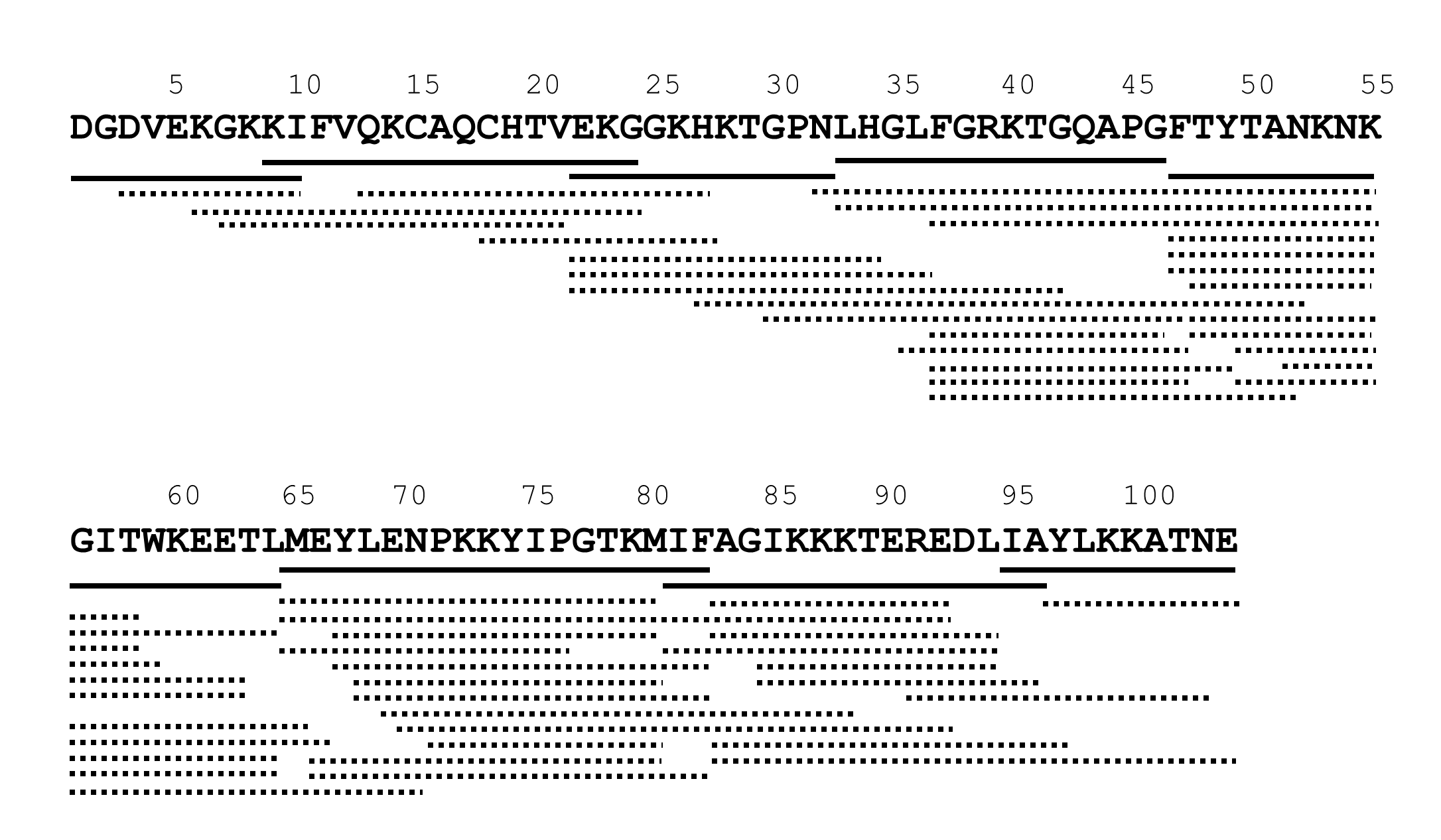
**

**Figure S-5.** The 55 peptides accounting for 100% coverage of the whole sequence

**Figure S-6.** (A) Deuteration level of the representative peptides in the specific regions of cyt *c*. Deuteration level at 10, 30, 100, 300, 1000, and 3000 s was presented individually and in different color corresponding to their percentage. (B) and (C) Ribbon diagram of cyt *c* containing heme, specific helixs and sites indicated.


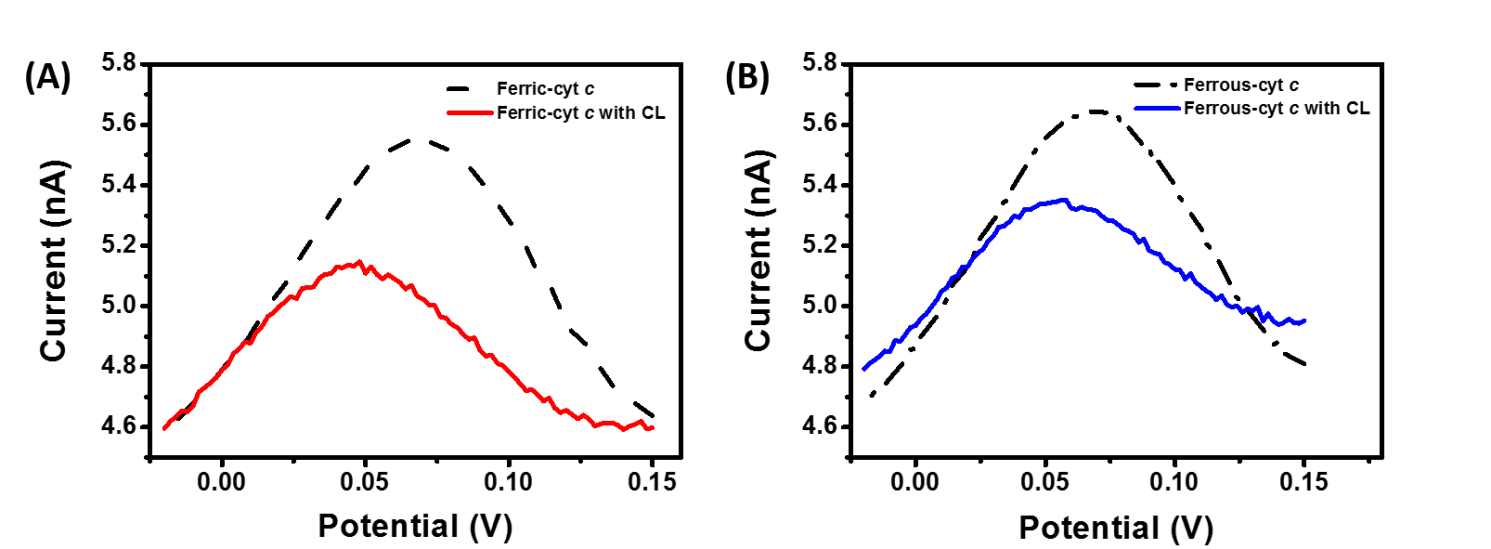


**Figure S-7.** AC voltammograms of ferric (A) and ferrous (B) cyt *c*-assembled gold electrodes. Black dashed voltammograms represented pristine cyt *c*. CL-bound ferric and ferrous cyt *c* was represented with red and blue, respectively.

**Preparation of cyt *c*-functionalized Electrode.** The gold disc electrode (2 mm diameter, CH Instruments) was polished with 0.05 µm alumina powder slurry to mirror smoothness and thoroughly rinsed with deionized water. Subsequently the electrode was activated by performing cyclic voltammetry (CV) between −0.4 and +1.6 V (vs. Ag/AgCl) for 20 cycles in 0.1 M H_2_SO_4_. To form self-assembled monolayers, the activated electrode was dipped into a mixture containing 3-Mercaptopropionic acid (MPA) and 6-Mercaptohexanol (MCH) for 16 h. Total concentration of the alkyl thiols was 5 mM (equilibrated in absolute ethanol) with a molar ratio of 1:3 (MPA:MCH). After being rinsed with deionized water and dried with nitrogen gas, the self-assembled electrode was immersed in a cyt *c* solution (50 µM equilibrated in 50 mM Tris-HCl buffer, pH 7.4) at 4 °C for 2 h. After being functionalized with cyt *c*, the electrode was rinsed with Tris-HCl buffer (50 mM, pH 7.4) and stored at 4 °C until electrochemical measurements.

**Electrochemical Measurements.** The electrode functionalized with either ferric or ferrous cyt *c* was incubated in CL/DPPC (230 μM) liposomal solution for 30 min, followed by a rinse using 50 mM Tris-HCl buffer to remove nonspecifically adsorbed liposomes. The interaction between ferric/ferrous cyt *c* and CL was studied in a three-electrode cell composed of a platinum wire as a counter electrode, an Ag/AgCl (3M KCl) reference electrode (CHI111, CH Instruments, Austin, TX), the functionalized gold disc as working electrode, and deaerated Tris-HCl buffer (50 mM, pH 7.0) as electrolyte by performing alternating current voltammetry (ACV) using an electrochemical workstation (CHI 660, CH Instruments, Austin, TX). The ACV parameters were 0.1 Hz in frequency, 25 mV in amplitude, and potential scanning over +0.25 − −0.15 V.
